# Inflammasome activation and formation of ASC specks in patients with juvenile idiopathic arthritis

**DOI:** 10.3389/fmed.2023.1063772

**Published:** 2023-03-01

**Authors:** Nico Wittmann, Neha Mishra, Jana Gramenz, Daniela Kuthning, Ann-Kathrin Behrendt, Lukas Bossaller, Almut Meyer-Bahlburg

**Affiliations:** ^1^Section of Pediatric Rheumatology, Department Pediatric and Adolescent Medicine, University Medicine, University of Greifswald, Greifswald, Germany; ^2^Section of Rheumatology, Department of Medicine A, University Medicine, University of Greifswald, Greifswald, Germany

**Keywords:** autoinflammation, inflammasome, apoptosis-associated speck-like protein (ASC), flow cytometry – methods, juvenile idiopathic arthritis

## Abstract

**Objective:**

The formation of large intracellular protein aggregates of the inflammasome adaptor ASC is a hallmark of inflammasome activation and characteristic of autoinflammation. Inflammasome activated cells release the highly proinflammatory cytokine IL-1β in addition to ASC specks into the extracellular space. Autoinflammatory activity has been demonstrated in systemic JIA, however minimal data exist on the role of inflammasomes in other JIA subtypes. We therefore investigated, if pyroptotic cells are present in the circulation of oligo- and poly-articular JIA.

**Methods:**

Peripheral blood of JIA patients (*n* = 46) was investigated for ASC speck formation, a key step in inflammasome activation, by flow cytometry and immunofluorescence. Free ASC and proinflammatory cytokine levels were determined by ELISA and multiplex assay.

**Results:**

Oligo-articular JIA patients showed a significantly increased proportion of ASC speck^+^ monocytes compared to poly-articular JIA patients. In serum free ASC alone is not sufficient to assess inflammasome activity and does not correlate with ASC speck^+^ monocytes. Compared to control several cytokines were significantly elevated in samples of JIA patients. JIA serum containing antinuclear antibodies, incubated with ASC specks boosts a secondary inflammation by IL-1β production in macrophages.

**Conclusion:**

For the first time, we detect *ex vivo* inflammasome activation by ASC speck formation in oligo- and poly-articular JIA patients. Most notably, inflammasome activation was significantly higher in oligo- compared to poly-articular JIA patients. This data suggests that inflammasome derived autoinflammation may have a greater influence in the previously thought autoimmune oligo-articular JIA patients.

## 1. Introduction

Over the last two decades autoinflammation has been more and more distinguished from autoimmunity. Back in 2006, McGonagle et al. (1) proposed an immunological disease continuum model of inflammation against self with “pure” adaptive and innate immune diseases at the opposite ends. Thereby, activation of the adaptive immune system results in autoimmunity, characterized by involvement of B- and T-cells and the production of highly antigen-specific autoantibodies. In contrast, uncontrolled activation of the innate arm of the immune system by neutrophils, monocytes or macrophages can cause autoinflammation. In monogenetic autoinflammatory diseases such as CAPS, FMF or PAPA the regulation of inflammasomes in monocytes and macrophages is disturbed, resulting in autoinflammation (2, 3). Inflammasomes are cytosolic multiprotein complexes, containing inflammasome sensors including AIM2/IFI16, NAIP/NLRC4, NLRP3/6/7 and a central adaptor protein called ASC (apoptosis-associated speck-like protein containing a caspase activating and recruitment domain). Upon activation the adaptor protein ASC is recruited into a multiprotein complex and assembles into large helical fibrils, known as ASC speck. Inflammatory caspases are then recruited, resulting in the activation of caspase-1 and cleavage of pro-IL-1β and pro-IL-18 into their biological active pro-inflammatory forms (4). Finally this process is leading to pyroptosis, an inflammatory form of cell death (5).

Juvenile idiopathic arthritis (JIA) is a complex autoimmune inflammatory disorder and the most common childhood chronic rheumatic disease. Currently, seven JIA subtypes can be distinguished mainly based on clinical criteria according to criteria of the International League of Associations for Rheumatology (ILAR): Oligo-articular (persistent and extended), Poly-articular rheumatoid factor (RF) negative and positive, psoriatic Arthritis (psJIA), Enthesitis related Arthritis (ERA), systemic Arthritis (sJIA) and the undifferentiated Arthritis (uJIA) (6, 7). JIA forms a continuum between classical autoimmune and autoinflammatory conditions. While sJIA is generally considered as an autoinflammatory disease, other JIA subtypes, like the psJIA and ERA, show a mixed pattern between autoimmunity and autoinflammation (8). The most common subtypes, the oligo- and poly-articular JIA, are considered as autoimmune diseases. These subtypes are often associated with autoantibodies. Anti-citrullinated protein antibodies (ACPA) or RF are detected in about 2% of JIA patients representing the poly-articular RF positive JIA subgroup, while antinuclear antibodies (ANA) are often present in oligo-articular JIA patients (8, 9).

Previous studies suggest an involvement of inflammasomes in JIA pathogenesis, however cell type specific inflammasome activation has not been thoroughly investigated so far (10, 11). Therefore, we recently optimized a protocol, from Sester et al. (12, 13), for flow cytometric detection of ASC specks *ex vivo* to directly determine cell type specific inflammasome activation in peripheral blood. This method is particularly suitable for pediatric patients, since only small volumes of blood can be collected.

Using this method, we analyzed the contribution of autoinflammatory activity in JIA patients by detection of ASC specks in peripheral blood monocytes. In addition, we determined free ASC, cytokine levels and the inflammatory response of macrophages exposed to sera of JIA patients and ASC specks *ex vivo* as indicators of inflammasome activation.

## 2. Materials and methods

### 2.1. Reagents

The following reagents and chemicals were used: ultrapure LPS, EDTA (Invitrogen, Waltham, MA, United States), albumin bovine fraction V (BSA) (Serva, Heidelberg, Germany), DMSO, EGTA, HEPES-KOH, MgCl_2_, nigericin (Nig), phenylmethylsulfonyl fluoride (PMSF), trypan blue (all Sigma-Aldrich, St. Louis, MO, United States), GlutaMAX™ Supplement (Thermo Fisher Scientific, Waltham, MA, United States), Cytofix/Cytoperm™ Fixation/Permeabilization Kit (BD Bioscience, Franklin Lakes, NJ, United States), fetal calf serum (FCS), Ficoll, HEPES, PBS, Penicillin/Streptomycin, RPMI1640 (all PAN-Biotech, Aidenbach, Germany). The following Antibodies (Abs) were used: rabbit-polyclonal anti-ASC (AL177, Adipogen, San Diego, CA, United States), anti-rabbit-IgG-AlexaFluor488 (Invitrogen, Waltham, MA, United States) and lineage-specific antibodies CD3-PerCP, CD4-APC, CD14-APC-Cy7, CD16-PE-Cy7 (Biolegend, San Diego, CA, United States).

Reagents for immunofluorescence microscopy were: bovine serum albumin (BSA) (Serva, Heidelberg, Germany), methanol (J.T. Baker, Phillipsburg, NJ, United States), sodium pyruvate, saponin (Sigma-Aldrich, St. Louis, MO, United States), Image-iT™ FX Signal Enhancer and ProLong™ Diamond Antifade Mountant with DAPI (Invitrogen) and the following Abs were used: rabbit anti-ASC (AL177), mouse anti-beta-actin (Sigma-Aldrich), anti-rabbit-IgG-AlexaFluor488 (Invitrogen), anti-mouse-IgG-AlexaFluor594 (Dianova, Hamburg, Germany).

### 2.2. Patient acquisition and blood sample processing

All healthy donors and patients were recruited at the University Medicine Greifswald and included in the study after written informed consent. The study was conducted in accordance with the Declaration of Helsinki, and the protocol was approved by the local ethics committee (BB 014/18; BB 158/19; BB 188/20; BB032/21). After coagulation was complete, the serum was centrifuged for 10 min at 1,000 *g*. Peripheral blood mononuclear cells (PBMCs) were isolated from blood using density gradient centrifugation as previously described (12).

### 2.3. Monocyte isolation

Monocytes were isolated by CD14^+^ selection using magnetic cell separation and CD14 MicroBeads (Miltenyi Biotec, Bergisch Gladbach, Germany) according to manufacturer instructions. Isolated monocytes were centrifuged and resuspended in CellGenix® GMP DC media (CellGenix, Freiburg Breisgau, Germany) with granulocyte-macrophage colony-stimulating factor (GM-CSF) (PeproTech, Hamburg, Germany).

### 2.4. Freezing and thawing cells

After isolation, PBMCs were centrifuged and re-suspended in FCS + 10% DMSO. PBMCs were frozen away at −80°C, using a Mr. Frosty™ freezing container (ThermoFisher Scientific) and transferred on the next day into the gas phase of a liquid nitrogen container. For thawing, cells were warmed up fast and washed twice with PBS supplemented with 2 mM EDTA.

### 2.5. Cell culture

Isolated monocytes were seeded 1 × 10^6^/ ml into a flat bottom cell culture plate with CellGenix® GMP DC media and GM-CSF (500 UI/ml) for 3 days. After macrophage differentiation, the supernatant was removed and RPMI medium containing 1 ng/ ml LPS was added. Priming was performed for 6 h. Then extracellular ASC specks and serum, with final dilution of 1:80, were added for a 24-h incubation. On the next day the supernatant was removed and frozen at −20°C until further usage. The monocytic cell line THP1-ASC-GFP was cultured in RPMI 1640 (Thermo Fisher Scientific) supplemented with 10% FCS; Gibco™ GlutaMAX™ Supplement; 10 mM HEPES; 1 mM sodium pyruvate (Sigma-Aldrich); and 1:100 Penicillin/Streptomycin (10.000 U/ml/10 mg/ml) at 37°C.

### 2.6. Production and purification of ASC specks

THP1-ASC-GFP cells were grown in T75 flasks, until they had reached density of 1 × 10^6^ cells/ ml. The THP1-ASC-GFP cells were centrifuge and the pellet was stored at −20°C. To make a large preparation, we used the pellet from at least 10 flasks. The cell pellets were lysed with CHAPS buffer and the *in vitro* assembly and purification of ASC specks was performed as described by Fernandes-Alnemri et al. (14). The presence of intact ASC specks and their purity was verified by immunofluorescence microscopy and flow cytometry. We used a MACS Quant10 (Miltenyi Biotec) to determine the amount of extracellular ASC specks in the solution.

### 2.7. ELISA

The serum samples were analyzed for their free ASC with a human PYCARD/ASC/TMS1 ELISA (LSBio, Seattle, WA, United States) according to manufacturer’s instructions. Supernatant from LPS primed macrophages incubated with extracellular ASC specks and diluted serum was used in a human IL-1β/IL-1F2 ELISA (R&D Systems, Minneapolis, MN, United States) according to manufacturer’s instructions. Plate measurement was performed on a tecan infinite F50 (Tecan, Männedorf, Switzerland).

### 2.8. Flow cytometry

Freshly isolated PBMCs were fixed with Cytofix™ (BD Bioscience) on ice according to the manufacturer instructions. After washing with the permeabilization medium Cytoperm™ (BD Bioscience), fixed samples were kept at 4°C in a permeabilization medium overnight. For longer storage of fixed cells, we recommend a washing step and storage of samples in PBS. Cells were stained and measured on the next day as previously described (12).

### 2.9. Immunocytochemistry and fluorescence microscopy

Shandon cytospin 4 (Thermo Scientific) was used to deposit thawed cells into a clearly defined area of a “SuperFrost® plus” glass slide (R. Langenbrinck, Emmendingen, Germany). Fixation was done with ice cold methanol at −20°C for 15 min. The glass slides were then stored at −80°C until further usage. For staining, glass slides were warmed up fast and staining was performed as described by Wittmann et al. (12).

### 2.10. Cytokine determination

Plasma- or serum samples were frozen and thawed for the analysis in a Legendplex (13-plex) Human Inflammation Panel 1 (Biolegend). Samples were measured in duplicates and incubated with beads at 4°C overnight and for 1 h at RT on the next day. The further work steps were performed according to the manufacturer instructions. Samples were measured on a FACS Canto II and evaluated with the LEGENDplex™ Cloud-based Data Analysis Software (Biolegend collaboration with Qognit). If the value was underneath the detection limit than the value was set equal to the lowest calculated standard value.

### 2.11. Statistical analysis

Statistics were calculated with Prism software (GraphPad, San Diego, CA, United States). Data are presented as the mean ± SEM from the number of assays indicated. Multiple comparisons were analyzed by a Kruskal–Wallis-Test, follow by Dunn’s multiple comparisons test. Detection of difference between two groups a Mann–Whitney-*U*-Test was performed as appropriate. A *p*-value of *p < 0.05, **p < 0.01, or ***p < 0.001 was considered significant.

## 3. Results

### 3.1. ASC speck formation in peripheral blood of JIA patients

All patients and healthy controls were recruited at the university medicine Greifswald and signed informed consent. We focused on the most common subtypes, the oligo- and poly-articular JIA. These patients were classified according to ILAR criteria and included into the study regardless of their therapy and disease activity (Table 1) (6). Since we were looking at the ASC speck status at an arbitrary time point, we decided to include follow-up samples and consider them as individual samples. The follow-up was on average after 5.4 months, regardless of whether the first value was ASC speck^+^ or not. A total of 46 samples from 29 different patients were included in the study. For ethical reasons it was not possible to recruit healthy children. Therefore, we acquired five adult healthy donors (HD) as reference for cell-based experiments. In addition, to have a reliable age-matched comparison group for serum analyses, we therefore used serum of 10 pediatric patients from a SARS-CoV2 antibody screening study. These patients were negative for SARS-CoV2 specific antibodies and did not suffer from any rheumatic diseases. Characteristics of control and patient samples are shown in Table 1.

**Table 1:** Characteristic of control and patient samples.

	Control groups		JIA			
Variable	Adult HD	Children donor	Total	Oligo-articular*	Poly-articular**	
No. of patients	5	10	46	25	21	
Sex. no. male/female	3/2	7/3	6/40	3/22	3/18	
Age. mean.	31.6	10.1	9.8	9.8	9.8	
(range) years	(25–42)	(5–16)	(3–17)	(3–17)	(3–17)	
SJC	n.a.	n.d.	0–7	0–7	0–2	
TJC	n.a.	n.d.	0–17	0–17	0–6	
ESR. mean.	n.d.	n.d.	19.2	16.2	23	
(range) mm/h			(2–84)	(2–50)	(2–84)	
CRP. mean.	n.d.	n.d.	6.8	4.9	9	
(range) mg/dl			(<3.1–50.2)	(<3.1–16.5)	(<3.1–50.2)	
ANA. no. of positive patients. (%)	n.d.	n.d.	36 (78.2)	21 (84)	15 (71.4)	
Platelet count.	n.d.	n.d.	327.5	311.2	348.9	
(range) in Gpt/l			(156–548)	(196–469)	(156–548)	
MPV.mean.	n.d.	n.d.	9.38	9.5	9.3	
(range) in fl			(8.1–11.8)	(8.1–11.7)	(8.7–10.8)	
Treatment. (No. of patients)	n.a.	n.d.		NSAID (3)	NSAID (3)	
				MTX (3)	MTX (6)	
				Biologics (10)	Biologics (4)	
				Biologics + MTX (6)	Biologics + MTX (4)	
					Other (1)	

*Persistent and extended; **RF negative and positive. ANA, antinuclear antibodies; CRP, C-reactive protein; ESR, erythrocyte sedimentation rate; fl, femtoliter; HD, healthy donor; JIA, juvenile idiopathic arthritis; MPV, mean platelet volume; MTX, methotrexate; n.a., not applicable; n.d., not determined; NSAID, non-steroidal anti-inflammatory drug; SJC, swollen joint count; TJC, tender joint count.

**Figure 1:**
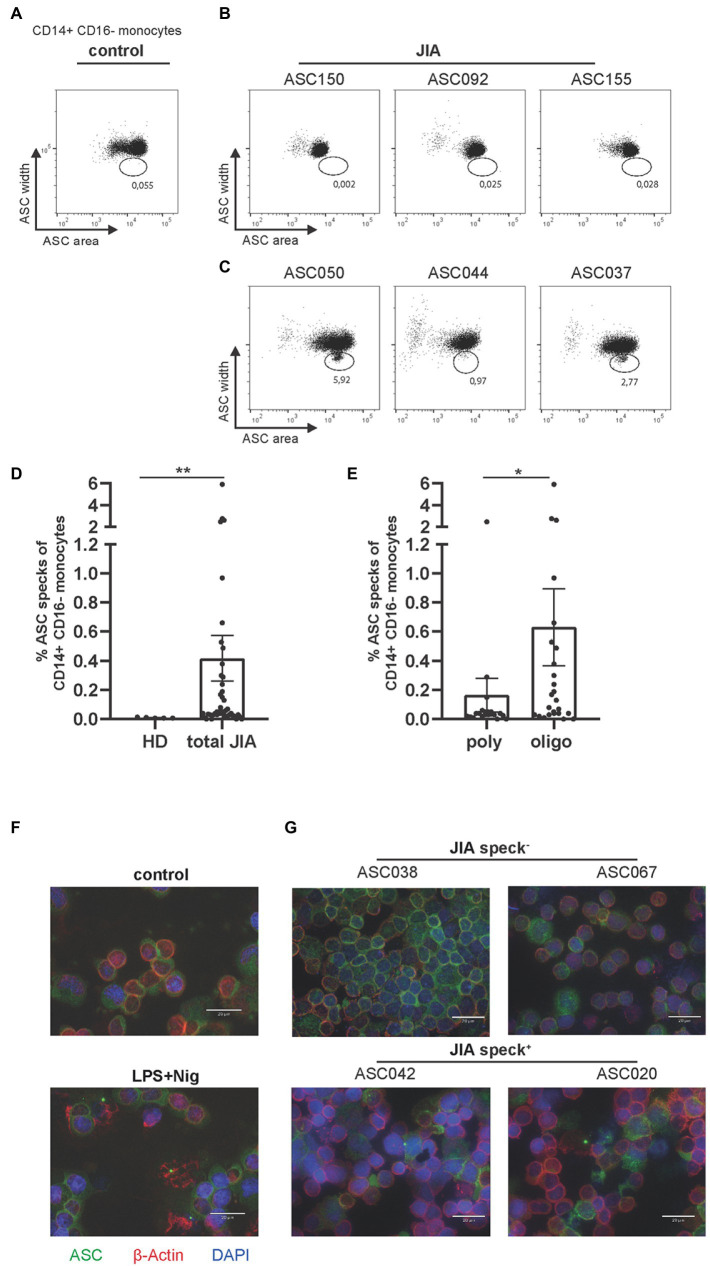
Detection of ASC specks in JIA patients, predominantly in the oligo-articular subtype. **(A)** Representative dot plot of CD14^+^CD16^−^ monocytes from healthy donor. **(B,C)** Representative dot plots of either ASC speck^−^ or speck^+^ CD14^+^CD16^−^ monocytes *ex vivo* from JIA patients. **(D)** Quantification of data from five adult HD and 46 JIA patient samples. **(E)** Quantification of JIA patients by subtype. **(F)** Representative fluorescence microscopic images of ASC speck formation in PBMCs from adult HD after stimulation with LPS and Nig or untreated and **(G)** representative patient samples. ASC (green), *β*-actin (red) and DAPI (nuclei, blue) (scale bar 20 μm). **(D,E)** Mann–Whitney-*U*-test was performed, shown mean ± SEM. A *p*-value *p < 0.05; **p < 0.01 was considered significant.

As a direct readout of inflammasome activity we determined ASC speck formation *ex vivo* by flow cytometry on a single cell level as recently published (12, 13). To obtain a reliable negative control, blood from five adult HD was used. One dot plot from an adult HD without ASC specks in CD14^+^ CD16^−^ monocytes is displayed (Figure 1A) and compared to representative ASC speck^−^ and ASC speck^+^ examples from JIA patients (Figures 1B,C).

Significantly higher numbers of ASC speck^+^ monocytes were detected in peripheral blood of JIA patients compared to the adult HD control group (Figure 1D). In particular, ASC speck^+^ monocytes were detected significantly more frequently in samples from oligo-articular compared to poly-articular JIA patients (Figure 1E). Although many poly-articular patients were ANA positive, a tendency toward more ASC speck^+^ monocytes was observed within JIA patients with an elevated ANA titer (Supplementary Figure S1). To further confirm the formation of ASC specks in a fraction of patient samples, we used widefield fluorescence microscopy. As a positive control LPS and nigericin (Nig) activated PBMCs were used and compared to resting cells (Figure 1F). In patient samples where ASC^+^ speck monocytes were found by flow cytometry, ASC specks were also clearly visible by widefield fluorescence microscopy. No ASC speck^+^ cells were detected under resting conditions, where ASC was homogeneously distributed over the cell. ASC specks in PBMCs from JIA patient’s *ex vivo* appeared similar, but in less quantity, to the ASC specks in LPS primed and Nig stimulated cells (Figure 1G).

We therefore conclude that ASC speck^+^ monocytes can be detected by flow cytometry and widefield fluorescence microscopy in peripheral blood of JIA patients, especially in oligo-articular patients but not in healthy adult donors.

**Figure 2:**
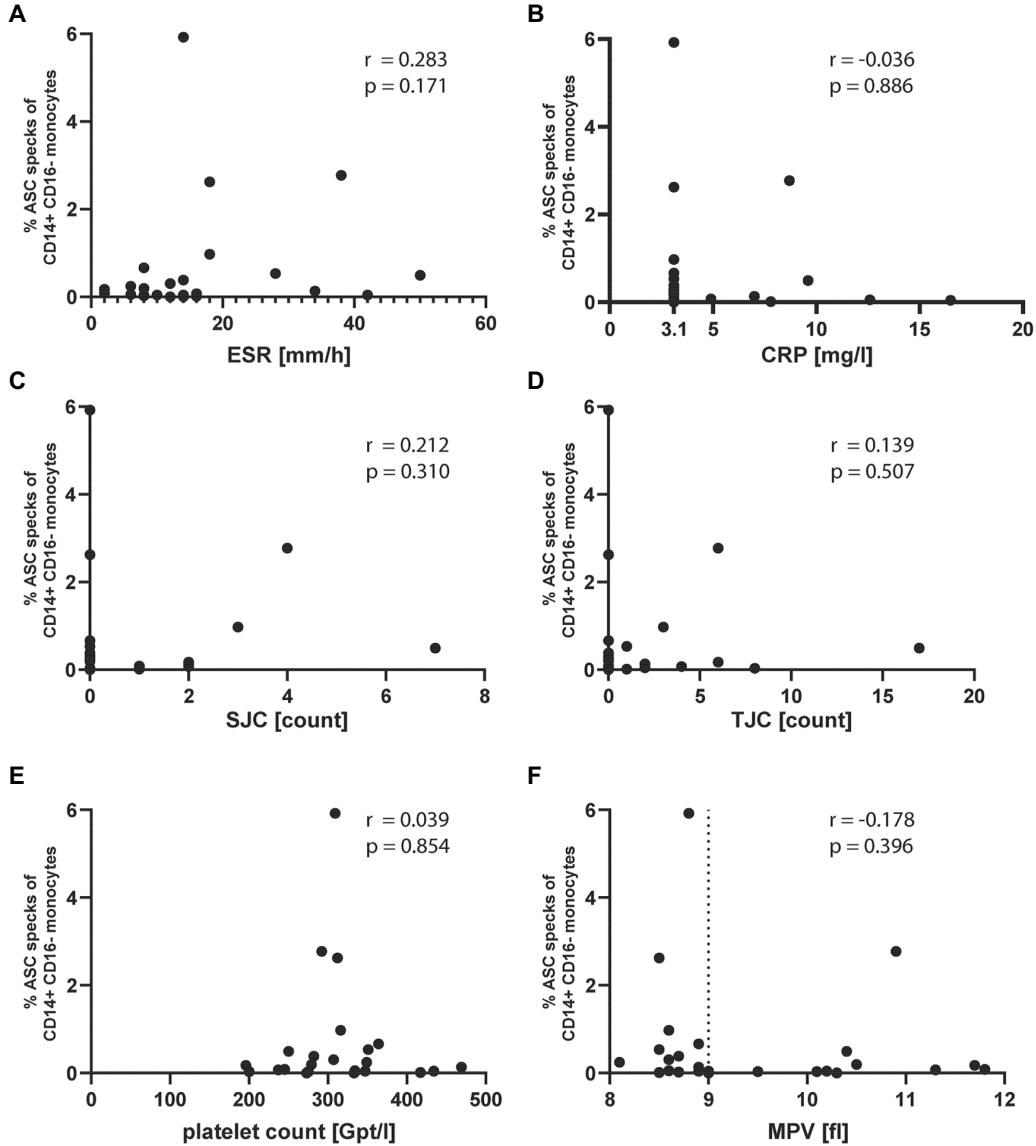
ASC specks in oligo-articular JIA patients do not correlate with common inflammatory markers or platelet count and size. Correlation of % ASC speck^+^ CD14^+^CD16^−^ monocytes in oligo-articular JIA patients with **(A)** ESR, **(B)** CRP, **(C)** SJC, **(D)** TJC, **(E)** platelet count, or **(F)** MPV. Spearman r correlation, a *p*-value *p* < 0.05 was considered significant.

The detection of ASC specks suggests the formation of inflammasomes. In the oligo-articular JIA patient samples, we neither detected a correlation of ASC specks with common inflammatory markers including ESR and CRP (Figures 2A,B) nor with the swollen joint count (SJC) and tender joint count (TJC) (Figures 2C,D). Platelets were recently shown to boost the inflammasome capacity of monocytes through NLRP3 transcription, thereby enhancing ASC oligomerization, caspase-1 activity, and IL-1β secretion (15). However, we found no correlation of ASC specks with platelet count or platelet size (Figures 2E,F). Although not significant, most patients with increased ASC speck^+^ had a reduced platelet size.

### 3.2. Detection of free ASC and inflammatory cytokines

Next, we wanted to investigate if surrogate parameters for inflammasome activity could be detected in plasma or serum of JIA patients. During pyroptosis ASC specks can be released from cells, remain active and accumulate in the extracellular space, where they promote IL-1β maturation (16). Therefore, free ASC from 31 JIA samples was detected by anti-ASC ELISA. Serum samples from three CAPS patients, which were in an inactive disease status under therapy with Canakinumab, and synovial fluid from four gouty patients were used as positive controls. Both CAPS and gouty patients were adults and samples were obtained from the Section of Rheumatology, University Medicine Greifswald. As control group, we used serum from five pediatric patients, with non-rheumatic diseases from an antibody screening study.

**Figure 3:**
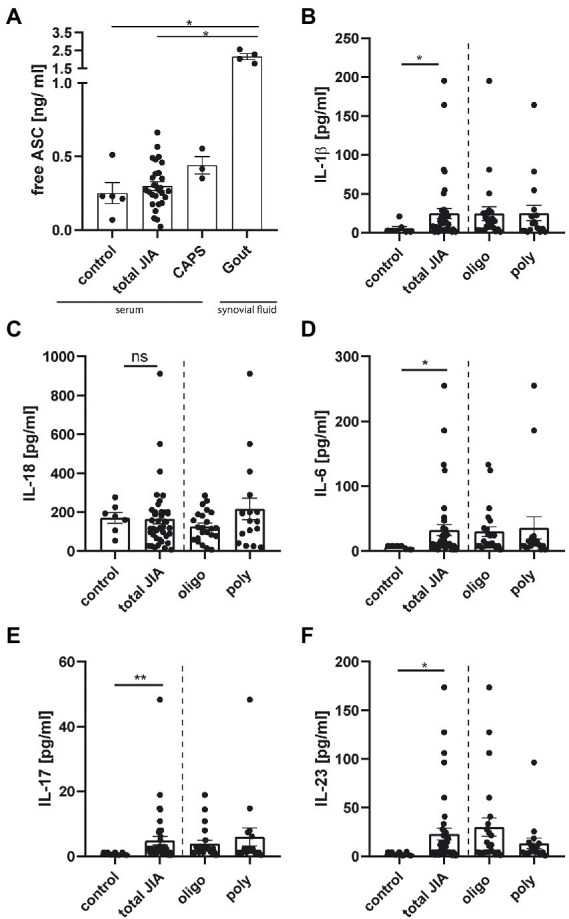
Cytokine levels, but not the amount of free ASC, are elevated in serum from JIA patients compared to control. **(A)** Quantification of free ASC in serum from five pediatric controls, three CAPS-, 31 JIA- patient samples and in the synovial fluid of four gout patients. **(B–F)** Quantification of cytokine levels from seven pediatric controls, and 41 JIA- samples measured in duplicates, represented as the mean from them. **(A)** Kruskal–Wallis test followed by Dunn’s multiple comparison test against control and **(B-F)** Mann–Whitney *U*-test were performed. A *p*-value *p < 0.05, **p < 0.01, or ***p < 0.001 was considered significant, shown mean ± SEM.

A broad range of free ASC concentrations was detected in JIA patients with some values similar to CAPS patients (Figure 3A). The synovial fluid of gouty patients showed significantly increased free ASC concentrations compared to the other groups, except the CAPS patients. Following inflammasome activation and ASC speck formation, caspase-1 is proteolytically activated and subsequently leads to the cleavage of pro-IL-1β and pro-IL-18 into their active forms. We therefore analyzed 41 serum samples from JIA patients for IL-1β, IL-18, as well as IFN-α2, IFN-γ, TNF-α, MCP-1 (CCL2), IL-6, IL-8 (CXCL8), IL-10, IL-12p70, IL-17A, IL-23 and IL-33. Serum from seven pediatric patients, with non-rheumatic diseases, from an antibody screening study served as control. Interestingly, a significant increase in the serum levels of IL-1β, but not IL-18 was detected in JIA samples compared to the control group (Figures 3B,C). This also applies to the cytokines IL-6, IL-17, and IL-23 (Figures 3D–F). No significant difference was detected for all other cytokines. A weak correlation was found between the two main inflammasome related cytokines IL-1β and IL-18 across all JIA patients (Supplementary Figure S2). Although a difference in the amount of ASC speck^+^ monocytes was detected, there was no significant difference in cytokine levels between the JIA subtypes. Our results indicate that the measurement of free ASC alone is not sufficient to draw conclusions about inflammasome activity *in vivo*. Several inflammasome associated cytokines are elevated in the JIA- compared to the control group.

### 3.3. Il-1β production from macrophages exposed to extracellular ASC specks is enhanced by serum of JIA patients with high titer of antinuclear antibodies

**Figure 4:**
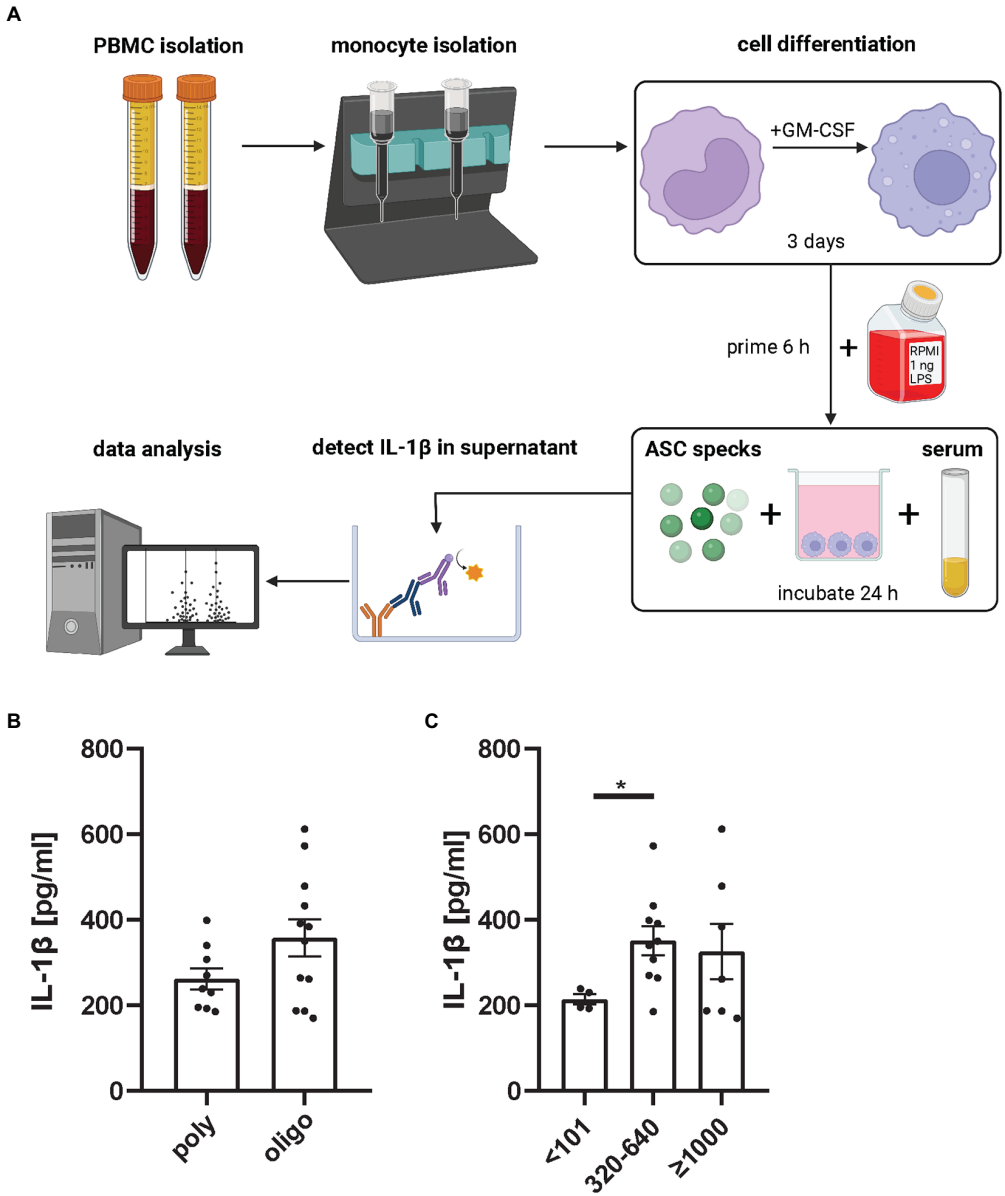
ANA^+^ JIA serum incubated with extracellular ASC specks causes primed macrophages to produce significant more IL-1β than ANA^−^ serum. **(A)** Monocytes from healthy donors were isolated, differentiated and primed with 1 ng LPS for 6 h and then incubated with ASC specks and patient serum for 24 h. IL-1β in supernatant was measured (18). Each dot in the bar graphs represents the mean of two independent experiments from 21 oligo- and poly-articular RF negative- JIA patients mean measured in two independent experiment represented as mean from them. Either grouped for their **(B)** subtype or their **(C)** ANA Titer. Mann–Whitney *U*-test was performed, shown mean ± SEM. A *p*-value *p < 0.05 was considered significant.

Autoantibodies have inflammatory properties as they can opsonize inflammatory material and thereby facilitate cellular uptake (16, 17). It is assumed that autoantibodies against ASC specks exist in autoimmune disease. Franklin et al. (16) demonstrated, that serum from autoimmune patients or lupus mice contains anti-ASC antibodies that were able to increase inflammation. In the previous section we had observed a tendency toward more ASC speck in monocytes from patients with high ANA serum titers. Thus, we next tested if autoantibodies from human JIA samples would enhance the secondary inflammation induced by extracellular ASC specks in macrophages. Therefore, monocytes of healthy donors were differentiated with GM-CSF into macrophages and stimulated with LPS. Primed macrophages were subsequently incubated with purified extracellular ASC specks and patient serum. As a readout of inflammasome activation IL-1β production was determined in the supernatant (Figure 4A).

We did not detect a difference in IL-1β production comparing ASC specks incubated with sera from poly-articular RF negative to oligo-articular JIA patients. Although sera from oligo-articular JIA patients showed a trend to induce a stronger secondary inflammation by macrophages in this assay (Figure 4B). Most importantly, there was a significant increase in IL-1β expression in ANA^+^ JIA samples compared to ANA^−^ JIA patients (Figure 4C). Thus, autoantibodies like ANA seem to favor a secondary inflammatory response to ASC specks by macrophages.

## 4. Discussion

In the current study we directly investigated inflammasome activation in peripheral blood of JIA patients. We used a recently optimized flow cytometry based method, that allows to determine cell type specific ASC speck formation *ex vivo* (12, 13). To our knowledge no study exists so far regarding *ex vivo* inflammasome activation in oligo- and poly-articular JIA patients. Moreover, only a few other publications investigate inflammasome activation by ASC speck formation in human blood (19–23). ASC specks were only detectable in CD14^+^CD16^−^ monocytes but no other PBMC subpopulation, indicating their role as important inflammasome activated cells.

Our data therefore show for the first time direct inflammasome activation by ASC speck formation *ex vivo* in peripheral blood of children with oligo- and poly-articular JIA. Surprisingly, ASC speck^+^ monocytes were detected with a significantly higher frequency in samples from oligo- compared to poly-articular JIA patients. Typically, oligo- and poly-articular JIA are considered as autoimmune disease. However, the classification of these subtypes in the autoinflammatory-autoimmunity continuum model is under current discussion. Szekanecz et al. (24) recently suggested a classification with oligo-articular JIA as a more autoinflammatory disease in comparison to poly-articular JIA. Our *ex vivo* data supports this suggestion.

In line with this idea, our data show a tendency between increasing ANA titer and greater numbers of ASC speck^+^ monocytes. This may be noteworthy for the current discussion of a new JIA categorization that comprises ANA^+^ JIA patients in one subgroup, independent of the number of affected joints (25). This new categorization encompasses ANA^+^ oligo- and poly-articular RF- JIA within the same group. Our data now support this idea of a JIA subtype classification based on ANA titers. In contrast, RF^+^ JIA patients form a separate group. In our small cohort (*n* = 5) we did not detect any ASC specks in the blood of poly-articular RF^+^ JIA patients. The detection of ASC speck^+^ monocytes might therefore be an additional indicator to distinguish between these subtypes, but this certainly has to be verified in a larger cohort.

Although our study does not define specific sensor protein upstream of inflammasome activation, this is the first report demonstrating ASC speck formation within a previously thought non-autoinflammatory disease such as oligo- and poly-articular JIA. Only little is known about the potential inflammasome sensor protein in JIA patients. In a Taiwanese study a genetic polymorphism was detected, whose carriers conferred an increased risk for oligo- and poly-articular JIA (26). Within these carriers the monocyte NLRP3 expression level can be increased. However, the hypothesis that NLRP3 might be the relevant sensor protein remains the focus of ongoing research.

A recently published study from Franklin’s group showed that platelets regulate monocyte innate immunity and identified platelet-derived p38 MAPK signaling as a critical trans-cellular regulator of cytokine production in monocytes (27). In our oligo-articular JIA cohort we did not detect a correlation between platelet quantity and number of ASC speck^+^ monocytes. Interestingly we observed, that most ASC speck^+^ patients have a reduced mean platelet volume (MPV). It is known that platelets are involved in inflammation and that a decreased platelet size is associated with platelet activation in SLE patients (28). Platelets can amplify arthritis and inflammatory proteins have been found in greater quantity in small platelets (29, 30). Possibly, smaller activated platelets could have a stronger immunomodulatory influence on monocytes and favor inflammasome activation in JIA patients. As we found no correlation of ASC specks with general inflammatory parameters, such as ESR or CRP, respectively, we currently assume that the pyroptotic cell death in CD14^+^CD16^−^ monocytes might be an independent marker of inflammation.

As an alternative readout for inflammasome activation we determined free ASC in the serum and observed a very diffuse distribution in the different patient groups. In our study, free ASC did not correlate with the number of ASC speck^+^ monocytes. This same observation has been made in a previous study in HIV patients, where we were unable to detect a correlation between free ASC in serum and ASC speck formation in monocytes. The lack of a correlation between free ASC and monocytic ASC specks may be due to a rapid clearance of circulating ASC specks by the reticuloendothelial system or other factors (19). Moreover, the detection of free ASC by ELISA does not allow conclusions regarding the multimerization status of ASC. In other words, it is unclear whether ASC detected by ELISA is assembled as monomer, oligomer or multimer. For these reasons, detection of free ASC from sera alone seems not to be sufficient to obtain a statement about inflammasome activation. Analyzing free ASC in synovial fluid and comparing it to ASC speck^+^ monocytes isolated from there might result in a different correlation. Free ASC in serum of CAPS patients, which were in complete remission under therapy, was not significantly elevated. This might be due to their therapy with Canakinumab resulting in an inactive disease status. As predicted, gout synovial fluid served as an excellent positive control, because the inflammatory response to monosodium urate crystals leads to a NLRP3 inflammasome formation, caspase-1 activation and IL-1β release (31).

While we saw a weak correlation between IL-1β and IL-18 in the JIA group, only IL-1β but not IL-18 was significantly elevated, compared to the control group. This is surprising since pro-IL-18 is permanently expressed in blood mononuclear cells and can be immediately processed into its biological active counterpart, while pro-IL-1β has to be synthesized first (32). In chronic inflammation macrophages produce IL-23, which induces the differentiation of naive CD4^+^ T cells into Th17 helper T cells. These cells are able to produce IL-6 and IL-17, but not IFNγ (33, 34). This consistent with our results, where significantly elevated cytokine levels for IL-6 and IL-17A, but not IFNγ were detected in JIA samples compared to controls. Higher IL-17A levels have been described earlier in oligo- and poly-articular patients during an active disease phase (35, 36). This cytokine promotes tissue inflammation and can attract monocytes, and other pro-inflammatory cells to the inflammatory site. The IL-23–IL-17 axis is already known to play an important role in JIA pathogenesis and other rheumatic diseases like PsA or ankylosing spondylitis (37). Similar to our data Yang et al. demonstrated that variant carriers, which increased risk of oligo-articular and poly-articular JIA, have an increased IL-17 response compared to control (26).

During pyroptosis the IL-1 family cytokines and ASC specks are released into the extracellular space. Extracellular ASC specks remain active and can propagate inflammation not only by maturating pro-IL-1β in the extracellular space but also by their phagocytosis by other cells. We have shown previously, that serum of autoimmune patients containing ANAs or of pristane-induced SLE mice boosts the IL-1β response of recipient cells (16). Interestingly, we now show, that ANA^+^ JIA sera induced a stronger secondary inflammation to extracellular ASC specks by the responding macrophages in cell culture. These data and previous studies suggest that ASC specks might be opsonized by autoantibodies and serve as an autoadjuvant thereby increasing the secondary inflammatory response (16, 17).

In conclusion, we clearly discovered direct inflammasome activity *ex vivo* in CD14^+^ CD16^−^ monocytes of oligo- and poly-articular JIA patients, but it was more prominent in oligo-articular JIA patients. In this first observational study we cannot assign what causes inflammasome activation in these patients. Further large cohort studies, including synovial fluid specimens, are necessary to verify these results. With regard to the autoimmunity-autoinflammatory continuum model, based on our findings, we do not consider oligo-articular JIA to be a solely autoimmune disease, but rather a mixed form. A better understanding of the role of inflammasomes and the interaction between the innate and adaptive immune system in oligo-articular JIA patients could lead to a better classification and subtype specific therapy.

## Data availability statement

The original contributions presented in the study are included in the article/Supplementary material, further inquiries can be directed to the corresponding author.

## Ethics statement

The studies involving human participants were reviewed and approved by Ethics Committee of the University Medicine Greifswald (BB 014/18; BB 158/19; BB 188/20; BB 032/21). Written informed consent to participate in this study was provided by the participants’ legal guardian/next of kin.

## Author contributions

NW, AKB, LB, and AM-B were involved in the conception and design of the study. NW, JG, DK, NM, and AKB performed the experimental work, collected and analyzed the data. NW, LB, and AM-B were involved in drafting the manuscript. LB and AM-B are the guarantors. All authors were involved in revising the manuscript, read and agreed to the published version of the manuscript.

## Funding

This research was supported by the University Medicine Greifswald, Germany and the Forschungsverbund Molekulare Medizin Greifswald (FOVB-2018-05 to A-KB); the European Regional Development Fund (EFRE, GHS-20-0031 to AM-B), and by German Research Foundation Grant (DFG BO 4325/3–1 to LB). NW was supported by the state graduate scholarship program, University Greifswald.

## Conflict of interest

The authors declare that the research was conducted in the absence of any commercial or financial relationships that could be construed as a potential conflict of interest.

## Publisher’s note

All claims expressed in this article are solely those of the authors and do not necessarily represent those of their affiliated organizations, or those of the publisher, the editors and the reviewers. Any product that may be evaluated in this article, or claim that may be made by its manufacturer, is not guaranteed or endorsed by the publisher.

## Supplementary material

This Supplementary material for this article can be found online at: https://www.frontiersin.org/articles/10.3389/fmed.2023.1063772/full#supplementary-material

Supplementary Figure S1 Increased amount of ASC speck^+^ monocytes in patients with higher ANA titer. Quantification of data from JIA patients. Mann–Whitney-U-test was performed, shown mean ± SEM. A p-value **p* < 0.05 was considered significant.

Supplementary Figure S2 Correlation of IL-1β and IL-18 in serum from JIA patients. Spearman r correlation, a p-value **p* < 0.05 was considered significant.
